# Wanted: A Sense of Asepsis

**Published:** 1912-08-15

**Authors:** John S. Marshall

**Affiliations:** Berkeley, Cal.


### WANTED: A SENSE OF ASEPSIS.

JOHN S. MARSHALL, M.D., SC.D., BERKELEY, CAL.

Have we, as practitioners of an important specialty of the healing art, a proper sense of asepsis? If a just opinion can be formed from observing the clinical teaching in our dental colleges, the methods employed in the offices of nearly all classes of dental practitioners and the demonstrations at the clinics of our state and national societies, then we must admit that we fall far short of a proper sense of asepsis.

What is asepsis? Asepsis from the surgical standpoint is that condition, maintained during the operation or treatment and the after process of healing or cure, which is the result of any process or method rendering the tissues to be operated on, the hands of the operators, the instruments, dressings, fluids, nedicaments and ligatures, which come in contact with them, *germ-free,* and maintaining this condition during the operation or treatment and the after-process of healing or cure.

It would be manifestly unjust to say that dental practitioners as a class are ignorant of the principles that underlie asepsis. That these principles are taught in the lecture-rooms of most of our dental colleges there is no doubt; but this teaching is then practically ignored by a very large majority of the students in their clinical work in the infirmaries. This is due in part to the fact that the clinic-rooms are poorly equipped with sterilizing apparatus and that the teachers, demonstrators and professors who oversee and direct the operations of the students are often exceedingly lax in their own technic and do not insist on a faithful carrying out of the didactic teaching on this subject. I have been astonished, yes, appalled, at the utter disregard of all nodern, scientific methods of asepsis to be found in many of our dental colleges, in the offices of numerous dental practitioners and particularly at the clinics of our state and national societies.

Let me particularize as to some of the things that I have seen in the infirmaries of our dental colleges:

1. In one college there were only two sterilizers in the operating-room and one in the extracting-room for the use of more than fifty students. It needs no extensive calculation to prove that this provision for sterilization was entirely inadequate, and as a result the majority of the students contented themselves with simply wiping their instruments on a towel, often a soiled one, before passing to the next patient, rather than wait for their turn at the sterilizers. Furthermore, I have seen nerve broaches that had been used in septic pulp-canals tossed back into the box without sterilization and used again on a patient without even being dipped in phenol or other sterilizing fluid. One towel was made to do duty for an entire clinical period lasting from two to three hours and sometimes for two to three patients. Demonstrators frequently passed from one patient to another, exa nining and criticizing the work of the students, but never thinking it necessary to even wash their hands before placing their fingers in the mouth of the next patient. But worst of all, I have seen a prominent teacher pass through his operating-room with a mouth-mirror in a waistcoat pocket, examining the work of his students and going from chair to chair without once washing his hands or cleansing the mouth-mirror. Verily we do need "a sense of asepsis" and in this case something more.

2. I have visited the offices of many dental practitioners in which there was no provision for sterilizing instruments, and the only cleansing that I saw these instruments receive was a simple washing in warm water. I have been invited to examine a case, and after carefully washing my hands and sterilizing them with alcohol, have been offered a soiled towel to dry them with.

An office may be fitted up in the most elaborate manner with enameled furniture, marble floors and wainscoting and zinc painted walls, but this will prove of no value if the more important matters of local and personal asepsis are neglected or ignored. The charge, often made by physicians, and doubtless true, that infectious diseases such as tonsilitis, diphtheria, tuberculosis and syphilis have been frequently transmitted from one patient to another by unclean and septic dental instruments is a shame and a reproach to modern dentistry, and shows the need of a "proper sense of asepsis."

3. I have witnessed at the public clinics given by various state societies and at the clinics of the National Dental Association the same disregard of aseptic methods as those already mentioned. In fact, the one thing most prominently and indelibly impressed on my mind at the last meeting of the National Association was the reckless disregard of asepsis in many of the clinics, both by the operator himself and those that he permitted to inspect the progressive steps of his operation and its completion. Twice in my professional life I have been a patient at the clinics of the National Dental Association, and I therefore speak with authority and much feeling when I say that I looked with fear and trembling at the dirty fingers thrust into my mouth by the interested spectators. But never more. Personally I have always declined to operate at one of these public clinics for the reason that the facilities for aseptic operations were so meager or absolutely wanting that I dared not risk the health or life of the patient, or my own reputation.

In my work as an examiner of dental surgeons for appointment to the dental corps of the U. S. Army, I have found a lamentable lack of a proper sense of asepsis, and many young men have failed in their practical tests by reason of this fact.

I have always considered it my duty to draw the line very sharply on the question of a proper sense of asepsis, for the reason that great harm may result from uncleanly methods of operating. The remedy for such methods lies with the teachers in our dental schools and with the clinicians at our state and national associations. When teachers of prominence and clinicians of high standing in the profession are indifferent to, or ignorant of, the principles of asepsis, it cannot be expected that the students and young practitioners will be impressed with its value or see the necessity for such painstaking and elaborate preparations to prevent infection. The fact that infection does not always take place when aseptic methods are ignored is no assurance that it will not take place in any case. Resistance to disease is not equal in all individuals, and the virulence of infective organisms is not always the same. Untold suffering and many deaths have been the result of neglect and ignorance of a proper sense of asepsis.

Much of the trouble experienced by so many dentists in the treatment of septic conditions of the teeth and mouth is the result of an imperfect knowledge of the principles of asepsis and a worse than bad technic.

I know of a physician who left the chair of a dentist of high reputation because of his fear of infection on observing the unscientific technic practiced by the dentist. The public generally is also becoming educated along these lines, and many individuals will not expose themselves to such grave dangers from infection at the hands of a careless or ignorant dentist or surgeon.

Is it not time that this question of asepsis in all dental operations should be taken up in a serious manner and that its principles be taught and the technic practiced in the most careful and scientific manner?

There is no longer any excuse for lax methods in asepsis, and the sooner the profession awakes to the fact, the better it will be for the reputation of the profession and the health of the public. *Wanted: A sense of asepsis.—Journal A. M. A.*, July 13, 1912.

